# Modification of Medium Composition for Enhancing the Production of Antifungal Activity from *Xenorhabdus stockiae* PB09 by Using Response Surface Methodology

**DOI:** 10.1155/2018/3965851

**Published:** 2018-06-12

**Authors:** Chirayu Sa-uth, Paweena Rattanasena, Angsumarn Chandrapatya, Prapassorn Bussaman

**Affiliations:** ^1^Department of Biotechnology, Faculty of Technology, Mahasarakham University, Maha Sarakham 44150, Thailand; ^2^Community Public Health Sub-Department, Department of Applied Sciences, Faculty of Science and Technology, Phranakhon Si Ayutthaya Rajabhat University, Ayutthaya 13000, Thailand; ^3^Department of Entomology, Faculty of Agriculture, Kasetsart University, Bangkok 10900, Thailand

## Abstract

*Xenorhabdus stockiae* PB09 bacterium has been shown to exhibit antifungal activity against several plant pathogens. To improve its efficacy, the optimization of the nutritional components in culture media was performed. The medium components that have significant effects on antifungal activity of *X. stockiae* PB09 were initially identified using a fractional factorial design. Response surface methodology and central composite design were then used to create a model for optimizing the levels of carbon, nitrogen, and mineral sources that maximize antifungal activity of *X. stockiae* PB09. After that, the suitable carbon, nitrogen, and mineral sources were selected and adjusted by the second-order polynomial regression model, which predicted that 98.62% of antifungal activity could be obtained when the medium contained sucrose, yeast extract, NaCl, and K_2_HPO_4_ at 3.24, 23.71, 5.46, and 2.73 g/L, respectively. Laboratory verification of this recipe resulted in the antifungal activity at 97.95% in the shake flask experiment after 48-hour cultivation, which was significantly 27.22% higher than that obtained by using the TSB medium. In addition, *X. stockiae* PB09 cultured in the verified recipe by using 5 L fermenter could effectively inhibit the mycelial growth of *Phytophthora* sp., *Rhizoctonia solani*, *Pythium* sp., and *Fusarium oxysporum*. This study demonstrated that the RSM and CCD were shown to be valuable tools for optimizing the culture medium that maximize the antifungal activity of *X. stockiae* PB09.

## 1. Introduction

Entomopathogenic nematodes of the genera *Steinernema* are effective biological control agents for a wide range of agricultural pests [1]. When *Steinernema* nematodes infest the insect hosts, they release their symbiotic bacteria *Xenorhabdus* spp., into hosts' haemocoels. Then, *Xenorhabdus* spp. bacteria cause septicemia and release digestive enzymes that kill and degrade the host within 48 h [1]. A variety of metabolites produced by *Xenorhabdus* spp. can destroy the insect's immune system [2] and inhibit the fungal and bacterial competitors [3, 4]. *Xenorhabdus* spp. has been found to damage several insect pests, such as *Spodoptera exigua* Hűbner (beet armyworm) [5], *Manduca sexta* Linnaeus (tobacco hornworm) [6], *Plutella xylostella* Linnaeus (diamondback moth), *Otiorhynchus sulcatus* Fabricius (black vine weevil), and *Schistocerca gregaria* Forskal (desert locust) [7]. In addition, *Xenorhabdus* spp. has also been used as a biological control agent against *Luciaphorus perniciosus* Rack, the mushroom mite that is endemic in Thailand [8–10]. The antimicrobial compounds produced by *Xenorhabdus* spp. are known to inhibit several fungal plant pathogens [1, 3, 4, 11, 12], and these compounds have been isolated and identified, including xenorhabdins [13], xenocoumacin [14], nematophin [15], and indole derivatives [16].

Antifungal activity of entomopathogenic bacteria has been found to differ qualitatively depending on the bacterial species and strains as well as their culture conditions. Nutrition in medium culture can play an important role in triggering and increasing the levels of secondary metabolite production. The quantity and type of essential nutrients are found to be the effective means for not only restricting the bacterial growth but also specifically affecting their metabolic and regulatory pathways [17]. Optimization of culture media for enhancing the production of antibiotic activity of *Xenorhabdus* spp. has been developed by using the response surface methodology (RSM) [18–20]. Despite the presence of complex metabolic interactions, RSM can be used to evaluate the relative significance of several factors that affect the bacterial pathways [21]. This method is often employed after the “vital few” controllable factors have been identified, and it then needs to find the factors that set the optimization of the responses. This method has successfully been applied in many areas of biotechnology such as lactic acid, antibiotic, and enzyme productions [22–24].

The aim of this study was to optimize the recipe of culture medium for maximizing the antifungal activity of *X. stockiae* PB09 by using stepwise optimization as follows: (1) screening the most effective original medium; (2) modifying that original medium by replacing its carbon and nitrogen sources with new ingredients that significantly affect the antifungal activity by using the one-factor-at-a-time approach; (3) optimizing all the significant ingredients, whereby a fractional factorial design (FFD) was initially employed to screen the most significant factors, the steepest ascent method was then carried out for the suitable concentrations, and, finally, a central composite design (CCD) of RSM was applied to search for the optimal medium compositions; and (4) scaling up the cultivation in batch fermentation. The results obtained from this study could be beneficial for further enhancement of secondary metabolite production by *X. stockiae* PB09, especially in the large-scale settings.

## 2. Materials and Methods

### 2.1. Bacterial Cultures

*Xenorhabdus stockiae* PB09 isolate was derived from the infective juveniles (IJs) of *Steinernema siamkayai* Stock, Somsook, and Reid, which was obtained from the Department of Agriculture, Ministry of Agriculture and Cooperatives, Thailand, using the method described by Kaya and Stock [25]. *X. stockiae* PB09 was maintained in phase I and used throughout the study. To ensure phase I, the bacteria were subcultured onto NBTA agar (consisting of peptone, 10 g/L; beef extract, 3 g/L; NaCl, 5 g/L; and agar, 15 g/L, and supplementing with triphenyltetrazolium chloride, 0.040 g/L, and bromothymol blue powder, 0.025 g/L) and incubated at 28°C in the dark for 2-3 days. *X. stockiae* PB09 in phase I could be distinguished from phase II by its adsorption of bromothymol blue and by formation of blue colonies on NBTA.

A seed culture of *X. stockiae* PB09 was prepared by inoculating a full loop of phase I colonies into a 250 mL flask containing 100 mL of nutrient broth, which was cultivated at 28°C on a rotary shaker at 200 rpm for 16–24 h in complete darkness to result in the optical density (600 nm) of approximately 2.

### 2.2. Measurement of Bacterial Biomass

The biomass of bacterial cells was measured by optical density (OD) value of the culture at 600 nm using a spectral photometer, and the dry cell weight (DCW) was calculated as described by Wang et al. [26]. The calibration curve was initially plotted between the dilutions of bacterial cell suspension and the obtained optical density values. A fixed volume of each dilution was centrifuged at 10,000 rpm for 20 min, and its cell pellet was dried at 110°C in a hot air oven for 48 h and finally weighted for converting the optical density value into the dry cell weight (g/L).

### 2.3. Assay of Antifungal Activity

Cell-free supernatants of *X. stockiae* PB09 were *in vitro* tested for their efficacy against *Phytophthora* sp. mycelia growth by using the poisoned food technique [11]. *Phytophthora* sp. was provided by the Biocontrol Research Unit, Mahasarakham University, and was cultured on potato dextrose agar (PDA) at 25°C for 5–7 days (*L* : *D* = 12 : 12 h). Its young mycelia at the edge of colonies were then punctured by sterile 6 mm cork borer to produce mycelial plugs. In the meantime, cell-free supernatant of *X. stockiae* PB09 was mixed with sterile, melting PDA that had been cooled down to approximately 50°C at 10% (v/v) in the final volume of 10 mL and then poured into each 9 cm Petri dish. After PDA mixed with cell-free supernatant of *X. stockiae* PB09 became solidified, each 6 mm mycelial plug was placed upside down onto the center of each plate. The experiment was performed in four replicates. All plates were incubated in an incubator at 30°C for 5 days and the diameter of fungal colony of each plate was measured. The inhibitory activity of cell-free supernatant of *X. stockiae* PB09 against *Phytophthora* sp. mycelial growth was calculated using the following formula: *I* = ((*C*−*T*)/*C*) × 100, where *I* is the percentage of mycelial inhibition, *C* is the radial growth in control group, and *T* is the radial growth in treatment group.

### 2.4. Selection of the Optimal Nutrient Medium

The media, including Luria Bertani broth (LB) (tryptone, 10.0 g/L; yeast extract, 5.0 g/L; NaCl, 10.0 g/L), tryptone soy broth (TSB) (tryptone, 17.0 g/L; soytone, 3.0 g/L; glucose, 2.5 g/L; NaCl, 5.0 g/L; K_2_HPO_4_, 2.5 g/L), and modified yeast extract broth (YSG) (glycerol, 5.0 mL/L; yeast extract, 5.0 g/L; 1 M MgSO_4_, 5.0 mL/L; (NH_4_)_2_SO_4_, 2.0 g/L; 1 M KH_2_OP_4_, 5.0 mL/L; 1 M K_2_HOP_4_, 5.0 mL/L; and 1 M Na_2_SO_4_, 10.0 mL/L) were used in comparative studies to find the optimal nutrient medium for maximizing the antifungal activity. These media were adjusted to the final pH at 7.5 by using 2 mol/L NaOH and 2 mol/L HCl. The seed culture of *X. stockiae* PB09 (10 mL) was then transferred to each of these media (100 mL) in 250 mL flasks and incubated in dark at 28°C on a rotary shaker at 200 rpm for 72 h. The sample (4 mL) was taken out every day and spun (10,000 rpm, 20 min, 4°C) using centrifuge MPW-380R. Then, 2 mL of each resulting culture broth was filtered using 0.22 *µ*m-syringe filters to obtain cell-free supernatant and stored at 4°C until required. The experiment was repeated in triplicates.

### 2.5. Selection of the Optimal Carbon and Nitrogen Sources

The one-factor-at-a-time approach was used to determine the optimum carbon and nitrogen sources for enhancing the antifungal activity of *X. stockiae* PB09. Various sources of carbon (glucose, fructose, sucrose, maltose, and glycerol) and nitrogen (tryptone, yeast extract, peptone, beef extract, and soybean meal) were individually used to replace the corresponding sources in the original TSB medium, while NaCl and K_2_HPO_4_ were kept unchanged. The antifungal activity of *X. stockiae* PB09 bacteria cultured in different media were determined after 48 h of cultivation at 28°C in dark using a rotary shaker at 200 rpm. The aliquots of the resulting culture broths were individually centrifuged and filtered to separate the bacterial cells from the supernatants which were used for bioassays. The supernatants were stored at 4°C until required.

### 2.6. Optimization Procedure

#### 2.6.1. Fractional Factorial Design (FFD)

This study used the FFD to identify the medium's ingredient that had significant effects on the antifungal activity. Three independent variables, carbon (sucrose), nitrogen (yeast extract), and mineral (NaCl and K_2_HPO_4_) sources, were included in the 2-level fractional factorial design. According to the FFD, each variable was prepared in two levels: −1 for low level and +1 for high level. The design matrix of the tested variables and the levels of each variable are shown in Table 1. The main effect analysis of the influencing factors is shown in Table 2. The FFD is based on the first-order model:

(1) $$\begin{array}{c} Y=\beta_{0}+\sum \beta_{i}X_{i}, \end{array}$$

where *Y* is the predicted response, *β*_0_ is a constant coefficient, *β*_*i*_ is the linear coefficient, and *X*_*i*_ is the level of independent variables. In this study, three variables were screened by 8 experimental runs in addition with four runs at the center point. Design-Expert software (version 7.1, Stat-Ease, Inc., Minneapolis, USA) was used to design the experiments. Each experiment was conducted in three replicates.

#### 2.6.2. Path of Steepest Ascent

The method of steepest ascent was used to move rapidly to the vicinity of the optimum response. The center point of the FFD was considered as the origin of the path. The direction of steepest ascent was parallel to the normal of the contour line of the response curve of the model (1). The experimental design and the corresponding response of steepest ascent are shown in Table 3.

#### 2.6.3. Central Composite Design (CCD)

Continued from the above studies, the central composite experimental design (CCD) was used to optimize the concentrations of three effective nutrients. The three independent variables (sucrose, yeast extract, and mineral) were used as main variables and designated as *X*_1_, *X*_2_, and *X*_3_, respectively. The minimum and maximum ranges of variables were used as shown in Table 4. For developing the regression equation, the tested factors were coded according to the equation below:

(2) $$\begin{array}{c} x_{i}=\frac{\left(X_{i}-X_{0}\right)}{\Delta X_{i}},\;i=1,2,3,\ldots ,n, \end{array}$$

where *x*_*i*_ is the coded value of an independent variable, *X*_*i*_ is the actual value of independent variable, *X*_0_ is the actual value of the *X*_*i*_ at the center point, and Δ*X*_*i*_ is the step change value.

The response (percentage of mycelial inhibition) was added to a second-order model in order to estimate its correlation with the independent variables. An empirical second-order polynomial model is shown in the following equation:

(3) $$\begin{array}{c} Y=\beta_{0}+\sum \beta_{i}X_{i}+\sum \beta_{ii}X_{i}^{2}+\sum \beta_{ij}X_{i}X_{j}, \end{array}$$

where *Y* is the predicted variable, *X*_*i*_ and *X*_*j*_ are input variables which influence the response variable *Y*, *β*_0_ is the intercept term, and *β*_*i*_, *β*_*ii*_, and *β*_*ij*_ are measures of the effects of variables *X*_*i*_, *X*_*i*_^2^, and *X*_*i*_ *X*_*j*_, respectively.

According to the central composite design, a 2^3^ full-factorial CCD for three independent variables (each at five levels with four axial points and six replicates at the center points) was employed to fit the second-order polynomial model, which clearly showed that optimization of medium constituents would require at least 20 experiments.

For regression and graphical analyses of the obtained data, the Design-Expert software (version 7.1, Stat-Ease, Inc., Minneapolis, USA) was used. By optimizing the second-order polynomial equation (3) based on the desirability criterion of maximum percentage of mycelial inhibition, the suitable combinations of independent variables could be achieved. Student's *t*-test for the estimated coefficients was also applied for statistical analyses. Moreover, statistical analysis of the model was performed by analysis of variance (ANOVA). Fisher's *F*-test (overall model significance) was used for determination of the second-order polynomial model equation, and the quality of the fit of regression model equation was given by the coefficient of determination, *R* ^2^.

### 2.7. Batch Fermentation in 5 L Fermenter

Batch cultures of *X. stockiae* PB09 (3 L) were carried out in 5 L fermenter (Biostat B®, B. Braun Biotech International, Germany). The fermenter was equipped with two six-blade disc impellers; pH probes (Mettler-Toledo GmbH®, Switzerland); and devices for adjustment of DO (Mettler-Toledo GmbH, Switzerland), temperature, and foam. The cultivation temperature was 28°C with agitation speed of 200 rpm and aeration rate of 2.5 L/min. The seed culture of *X. stockiae* PB09 was transferred to 3 L of sterile medium (the optimized medium) in the fermenter at the ratio of 10% (v/v). The pH profile was adjusted by using 2 mol/L NaOH and 2 mol/L HCl. The batch fermentation process was performed for 72 h. The sample (5 mL) was taken out every 3 h, and then, 5 mL of the culture broth was spun (10,000 rpm, 20 min, 4°C) using centrifuge MPW-380R and filtered using 0.22 *µ*m-syringe filters to obtain cell-free supernatant, which was stored at 4°C until required. Four plant pathogenic fungi, namely, *Phytophthora* sp., *Pythium* sp., *Rhizoctonia solani* Kuhn, and *Fusarium oxysporum* Schlechtendahl, were used to determine the reduction of mycelial growth using the optimum medium obtained above. The experiment was repeated in triplicates.

## 3. Results and Discussion

### 3.1. Effects of Different Media on Biomass and Antifungal Activity of *X. stockiae* PB09

The effects of three different media, TSB, LB, and YSG, on biomass and antifungal activity of *X. stockiae* PB09 cells after cultivation by using shake flasks at different periods of time are shown in Table 5. The maximum dry cell weight was also found when the bacteria were cultured for 48 h on TSB (11.90 g/L). The cell-free supernatant of *X. stockiae* PB09 cultivated by using TSB medium for 48 h exhibited the highest inhibitory activity against the mycelial growth of *Phytophthora* sp. (70.73%). Therefore, TSB medium was shown to be the optimum medium for maximizing biomass and antifungal activity of *X. stockiae* PB09.

### 3.2. Effect of Various Carbon and Nitrogen Sources on Antifungal Activity of X. stockiae PB09

*Xenorhabdus* bacteria are potential producers of secondary metabolites with antifungal activity. The carbon and nitrogen are the important nutritional components in the medium for bacterial cultivation, and *Xenorhabdus* spp. bacteria have been shown to require both of these components for their metabolite production. Different species of *Xenorhabdus* bacteria seem to require rather different types of carbon and nitrogen sources, for example, glucose and peptone were the best carbon and nitrogen sources for antibiotic production by *Xenorhabdus nematophila* TB [19]; however, *Xenorhabdus bovienii* was found to be greatly influenced by glycerol and soytone [20]. In addition, tryptone and dextrose were shown to significantly increase the protease production by *Xenorhabdus indica* KB-3 [27].

**Figure 1:**
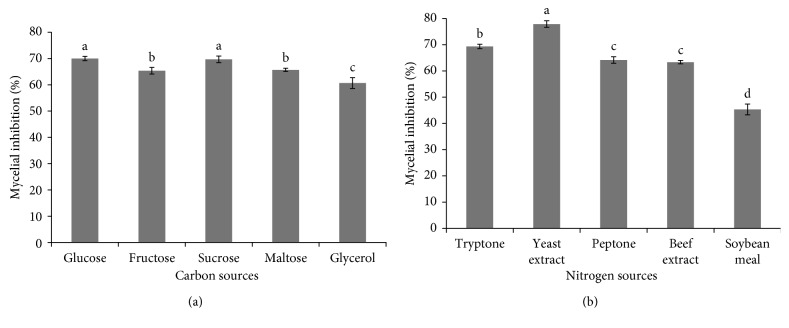
Effect of various carbon (a) and nitrogen (b) sources in the TSB medium on antifungal activity of *X. stockiae* PB09.

TSB medium was used as a base medium for selecting the alternative carbon and nitrogen sources, whereby both of which were maintained at the concentrations of 2.5 and 20 g/L, respectively (Figure 1). The effect of different carbon and nitrogen sources on *X. stockiae* PB09 antifungal activity is shown in Figure 1. The results showed that glucose could maximally enhance the antifungal activity, followed by sucrose, but both of which were not significantly different. Similar results were obtained in the study of Zhang et al. [28] that found the highest production of active antifungal substances by *Streptomyces hygroscopicus* BS-112 when glucose was used as a carbon source. In addition, Song et al. [29] also reported that glucose was the optimal carbon source for production of antifungal substances by *Brevibacillus laterosporus.*

For nitrogen sources, the highest antifungal activity could be achieved when using yeast extract (Figure 1). Furthermore, yeast extract was found to be the optimal nitrogen source for production of antifungal substances by *Mycena leptocephala* [30]. In addition, *M. leptocephala* was found to have increased biomass and antifungal activity when malt extract plus glucose was used as the carbon source and yeast extract was used as the nitrogen source. However, Elibol [21] reported that sucrose, glucose, yeast extract, and peptone had the most profound effects on actinorhodin production by *Streptomyces coelicolor* A3 (2) in complex medium. Therefore, in this study, sucrose and yeast extract were selected for further maximizing the antifungal activity of *X. stockiae* PB09.

### 3.3. Significant Factors

For the first optimization step, the FFD was used to screen the relatively significant variables for the production of antifungal activity. The effects of the medium components, including sucrose (*X*_1_), yeast extract (*X*_2_), and mineral (*X*_3_), on antifungal activity were determined. The experimental design and the FFD results are shown in Table 1. The values of the regression coefficients were calculated, and an equation of the first-order model could be written from the coefficients:

(4) $$\begin{array}{c} Y=74.44+3.55X_{1}+5.70X_{2}+2.61X_{3}, \end{array}$$

where *Y* was the percentage of mycelial growth inhibition, and *X*_1_, *X*_2_, and *X*_3_ are coded values of sucrose, yeast extract, and mineral, respectively.

Regression analysis of the FFD in Table 2 shows that sucrose, yeast extract, and mineral in the concentration range test had significant effects on antifungal activity (*P* < 0.05). Therefore, these three variables were used for further optimization experiment by using RSM.

### 3.4. The Steepest Ascent Path Test Results of Significant Factors

The path of the steepest ascent was used to attain the proper direction of changing variables, that is, increasing of sucrose, yeast extract, and mineral (NaCl and K_2_HPO_4_) concentrations, to improve the antifungal activity. The direction of the steepest ascent path was determined by using the first-order model (4). The experimental design and corresponding results of the steepest ascent were listed in Table 3. When considering the results from the path of steepest ascent, it was clearly seen that the percentage of mycelial inhibition showed the maximum of 90.50% at run number 2 (Table 3). Thus, the combination of run number 2 was selected as the center point of CCD.

### 3.5. Central Composite Experimental Results

This experiment was designed to optimize the concentrations of three selected variables in order to maximize the antifungal activity of *X. stockiae* PB09 by using the CCD of RSM. The three significant independent variables, that is, sucrose, yeast extract, and mineral (NaCl and K_2_HPO_4_) from the FFD were selected for designing the matrix. The design matrix of the tested variables is shown in Table 6.

By applying multiple regression analysis on the experimental data, the following second-order polynomial equation was used to explain the antifungal activity:

(5) $$\begin{array}{c} Y=96.06+2.71X_{1}+4.67X_{2}+2.69X_{3}+2.25X_{1}X_{2}-0.62X_{1}X_{3}-0.14X_{2}X_{3}-4.29X_{2}^{1}-4.37X_{2}^{2}-3.69X_{2}^{3}, \end{array}$$

where *Y* is the percentage of mycelial inhibition (%), and *X*_1_, *X*_2_, and *X*_3_ are the code values of sucrose, yeast extract, and mineral, respectively.

The results of response surface quadratic model in the form of analysis of variance (ANOVA) are shown in Table 7. This model was shown to be very significant by using Fisher's *F*-test (*F*_model_, mean square regression/mean square residual = 16.93) with a very low probability value ((*P*_model_ > *F*) < 0.0001). By determining the coefficient (*R* ^2^), the goodness of fit of the model was investigated. In this case, the value of the *R* ^2^ (0.9384) for (5) suggested that the sample variation of 93.84% for antifungal activity was the result of variables, and only 6.16% of the total variations was not explained by the model. High level of adjusted determination coefficient (adj. *R* ^2^ = 0.8830) also indicated the strong significance of the model. In addition, the good predictability of the model could be suggested by its statistically insignificant lack of fit ((*P*_model_ > *F*) = 0.3149). Moreover, the low value of coefficient of variation (CV = 3.14%) suggested that these experiments were precise and reliable.

**Figure 2:**
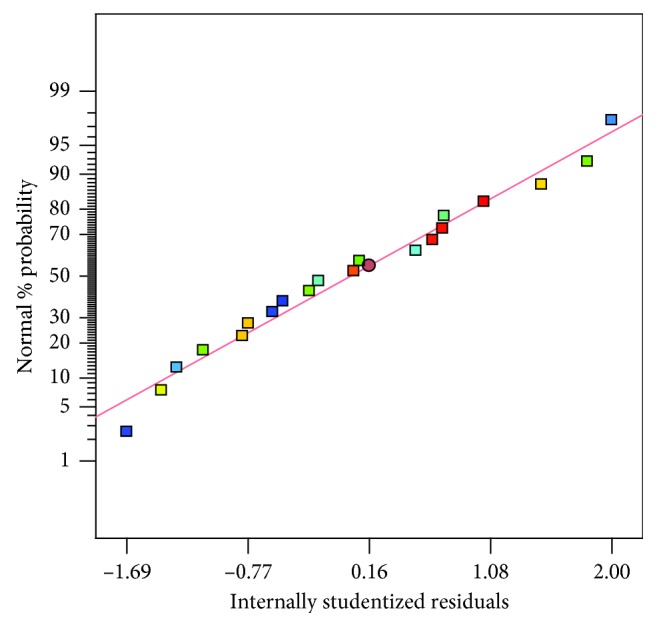
Normal plot of the residuals.

**Figure 3:**
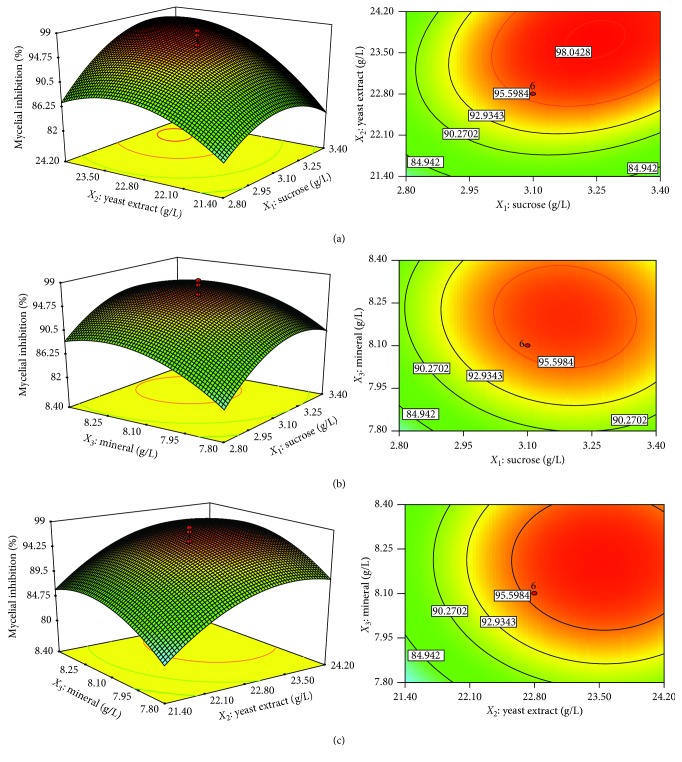
Response surface plots of the antifungal activity of *X. stockiae* PB09: (a) the effects of sucrose and yeast extract on antifungal activity; (b) the effects of sucrose and mineral on antifungal activity; (c) the effects of yeast extract and mineral on antifungal activity.

The normal probability plot of residuals was the most significant diagnostic method for the model and therefore set as default (Figure 2). To indicate whether there were no signs of problems in the data, the normality in the error term could be verified by linear patterns [23, 31]. All in all, for prediction within the range of selected variables, this model was considered to be suitable. The results obtained from experimental mycelial inhibition were compared with that of the statistical model (5) and shown in Figure 3, which indicates that the empirical model gave predicted data of the response in the same direction with the experimentally obtained data.

The 2D contour plot and the 3D response surface curve (Figure 3) were the graphical representations of the regression equation, which presents the effect of two variables on the antifungal activity of *X. stockiae* PB09 when maintaining the other two variables at their respective zero level. The significant interactions between the independent variables were indicated by the elliptical nature of the contour plots.

The 2D contour plot and 3D response surface curve are drawn in Figures 3(a)–3(c), according to the combined effects of sucrose-yeast extract, sucrose-mineral, and yeast extract-mineral, respectively, from which the percentage of mycelial inhibition for different concentrations of the variables could be predicted. Here, the response surface plots helped to understand the interactions between two nutrients and therefore to locate their optimum levels. The 2D contour plot of sucrose and yeast extract suggested their remarkable interaction, whereby the 3D response surface curve clearly indicated the sucrose and yeast extract concentrations (Figure 3(a)). From these results, the significant interactions between sucrose and yeast extract could imply that the ratio between *C* and *N* was critical for the production of active substances. The ratio between *C* and *N* was found to directly influence the levels of bacterial biomass and accumulation of their metabolites [23]. Interestingly, the previous study showed that the *C*/*N* ratio of optimized medium was lower than that of nonoptimized medium, and this ratio might be suitable for accumulation of active antifungal substances [23]. Nonetheless, the abovementioned interactions could be even more complex; therefore, the RSM was applied for further medium optimization. These plots (Figures 3(a) and 3(b)) represented that the optimal sucrose concentration was about 3.2 g/L. However, the relatively circular nature of the contour curves suggested that sucrose (Figure 3(b)) had no effect on minerals and showed that the optimal concentration of yeast extract was around 24.0 g/L. Also, the optimal value of minerals (Figures 3(b) and 3(c)) was around 8.2 g/L.

### 3.6. Validation of the Experimental Model

**Figure 4:**
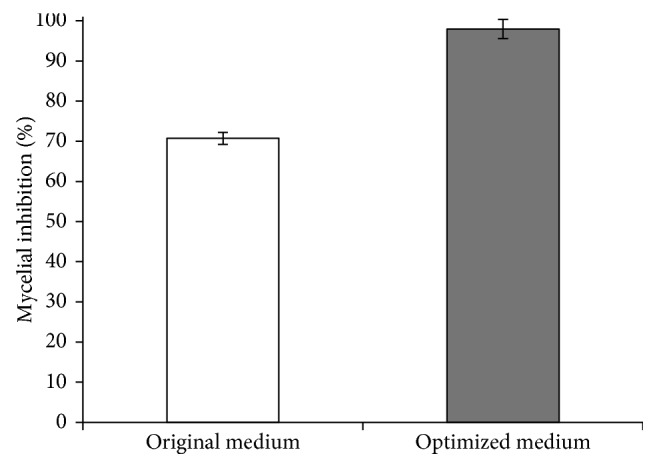
Antifungal activity of *X. stockiae* PB09 in the original medium and optimized medium.

By applying the regression analysis in (3), the prediction of optimum levels of sucrose, yeast extract, and mineral (NaCl and K_2_HPO_4_) could be achieved. The optimal values of evaluated components in coded units were as follows: *X*_1_ = 0.464, *X*_2_ = 0.647, and *X*_3_ = 0.313 with the corresponding *Y* = 98.62%, and their actual values were 3.24 (g/L) sucrose, 23.71 (g/L) yeast extract, and 8.19 (g/L) mineral (NaCl :K_2_HPO_4_ at 2 : 1). Consequently, the recipe of medium optimized by statistical approach was then evaluated by shake flask experiments. The maximum experimental antifungal activity was 97.95 ± 2.63%, which was similar to that of the predicted value (Figure 4). Therefore, this developed model was considered to be precise and reliable for predicting the production of antifungal activity by *X. stockiae* PB09. As the result of optimization, the antifungal activity could be enhanced by 27.22%, when compared to the original TSB medium (70.73 ± 1.48%) (Figure 4).

By applying this optimal medium for cultivation of *X. stockiae* PB09, the significance of linear and quadratic effects of sucrose, yeast extract, and mineral (NaCl and K_2_HPO_4_) was found. This may suggest that sucrose, yeast extract, and mineral (NaCl and K_2_HPO_4_) had direct influence on antifungal activity. The previous study has suggested that the production of microbial secondary metabolites could be significantly affected by even the small manipulations in the culture medium composition [32]. The RSM has been applied as the statistical tool for the studies of the influence of medium constituents, which results in the increase of antibiotic production in several antibiotic discovery programs. For instance, Wang and Liu [23] applied the RSM approach for optimizing the medium for *Paenibacillus* sp. to produce antifungal active substance and reported the 3.05-fold increase in antifungal activity when compared with the basal medium. Moreover, Zhang et al. [28] reported that a 2.8-fold increase of antifungal production by *Streptomyces hygroscopicus* BS-112z could be accomplished by using the RSM approach.

### 3.7. Effect of the Optimized Medium in Batch Fermentation

**Figure 5:**
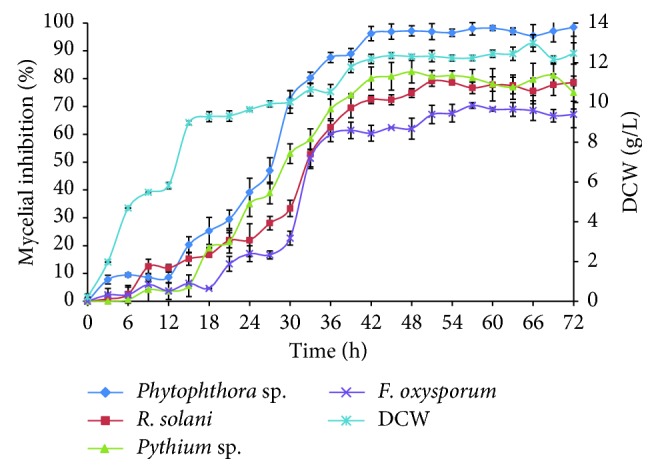
Levels of dry cell weight (DCW) and antifungal activity of *X. stockiae* PB09 in the optimized medium. The error bars in the figure indicate the standard deviations from three replicates.

To further determine the possibility of the regression models, the predicted optimal medium composition was evaluated by 5 L scaled fermenter.  Figure 5 showed the batch profile of dry cell weight (DCW) and antifungal activity of *X. stockiae* PB09 cultivated by using the optimal medium in 5 L fermenter. The antifungal activity increased rapidly during the first 42 h and remained stable until 72 h. After 72 h of incubation, *X. stockiae* PB09 was found to produce the antifungal substances that had strong *in vitro* inhibition against *Phytophthora* sp. (98.47 ± 0.83), *R. solani* (79.17 ± 1.93), *Pythium* sp. (82.50 ± 4.05), and *F. oxysporum* (70.37 ± 1.01) (Figure 5). This was corresponding to the previous studies that *Xenorhabdus* spp. was capable of producing the antifungal substances which inhibit several fungal plant pathogens, including, *Phytophthora nicotianae*, *Erwinia amylovora* [33], *Phytophthora capsici* [11], *Phytophthora cactorum*, *Fusicladium effusum* [12], *R. solani*, *Pythium* sp., and *F. oxysporum* [1].

In addition, the maximum dry cell weight of *X. stockiae* PB09 at 12.19 ± 0.24 g/L was obtained after 42 h of incubation. Therefore, this indicated that the optimized medium could improve the production of antifungal substances by *X. stockiae* PB09 in the large scale. Remarkably, high levels of antifungal activity and biomass of *X. stockiae* PB09 could be achieved in 5 L fermenter faster than that in the shake flask scale. This may be due to the addition of different neutralizing agents into various voluminal bioreactive systems. Similar results were obtained in the study of Wang and Zhang [34] and Wang et al. [26] that reported the strong influence of aeration and agitation on biomass and antibiotic production by *X. nematophila* YL001 in fermenter scale.

## 4. Conclusions

In this study, the response surface method (RSM) and central composite design (CCD) with the second-order polynomial model were shown to be valuable tools for optimizing the culture medium that maximize the antifungal activity of *X. stockiae* PB09. The optimal culture could enhance the antifungal activity from 70.73% to 97.95% in the shake flask experiments, which was very close to the predicted level at 98.62%. An overall 27.22% increase in antifungal activity was obtained as compared with TSB medium. Furthermore, *X. stockiae* PB09 cultured in the 5 L fermenter could effectively inhibit the mycelial growth of *Phytophthora* sp., *R. solani, Pythium* sp., and *F. oxysporum*.

## Figures and Tables

**Table 1:** Coded levels and real values for the experimental design and results of FFD.

Run	*X*_1_: sucrose (g/L)	*X*_2_: yeast extract (g/L)	*X*_3_: mineral (g/L)	Mycelial inhibition (%)	
				Observed	Predicted
1	−1 (1)	−1 (16)	−1 (5.5)	60.67	62.57
2	+1 (4)	−1 (16)	−1 (5.5)	70.67	69.67
3	−1 (1)	+1 (24)	−1 (5.5)	75.27	73.97
4	+1 (4)	+1 (24)	−1 (5.5)	80.67	81.07
5	−1 (1)	−1 (16)	+1 (9.5)	65.27	67.80
6	+1 (4)	−1 (16)	+1 (9.5)	78.33	74.90
7	−1 (1)	+1 (24)	+1 (9.5)	82.33	79.20
8	+1 (4)	+1 (24)	+1 (9.5)	82.27	86.30
9	0 (2.5)	0 (20)	0 (7.5)	76.34	78.35
10	0 (2.5)	0 (20)	0 (7.5)	80.27	78.35
11	0 (2.5)	0 (20)	0 (7.5)	79.45	78.35
12	0 (2.5)	0 (20)	0 (7.5)	77.33	78.35

**Table 2:** FFD regression results for antifungal activity.

Variables	Regression analysis		
	Coefficient	*F* value	Significant level
Intercept	74.44	15.99	0.0016^*∗*^
*X*_1_: sucrose (g/L)	3.55	11.64	0.0113^*∗*^
*X*_2_: yeast extract (g/L)	5.70	30.00	0.0009^*∗*^
*X*_3_: mineral (g/L)	2.61	6.31	0.0402^*∗*^

*R* ^2^ = 0.8725; ^*∗*^significant at *P* value less than 0.05.

**Table 3:** Steepest ascent experiment design.

Run Δ	*X*_1_: sucrose (g/L)	*X*_2_: yeast extract (g/L)	*X*_3_: mineral (g/L)	Mycelial inhibition (%)
Step length	0.3	1.4	0.3	
Base point	2.5	20.0	7.5	76.50

*Experiments*
Number 1	2.8	21.4	7.8	79.17
Number 2	3.1	22.8	8.1	90.50
Number 3	3.4	24.2	8.4	81.67
Number 4	3.7	25.6	8.7	67.50
Number 5	4.0	27.0	9.0	42.50

**Table 4:** Experimental ranges and levels of the independent variables.

Variable	Parameter	Ranges and levels				
		−1.68	−1	0	1	1.68
*X*_1_	Sucrose (g/L)	2.60	2.80	3.10	3.40	3.60
*X*_2_	Yeast extract (g/L)	20.45	21.40	22.80	24.20	25.15
*X*_3_	Mineral (g/L)	7.60	7.80	8.10	8.40	8.60

**Table 5:** Effect of different media on biomass and antifungal activity of *X. stockiae* PB09 against *Phytophthora* sp. after cultivation by using shake flasks at different periods of time.

Media	Biomass (g, dry cell weight/L ± SD)			Mycelial inhibition (%, mean ± SD)		
	24 h	48 h	72 h	24 h	48 h	72 h
TSB	9.78 ± 0.16^a^	11.90 ± 0.43^a^	11.79 ± 0.14^a^	63.18 ± 4.03^a^	70.73 ± 1.48^a^	70.73 ± 2.57^a^
LB	8.14 ± 0.11^b^	10.12 ± 0.11^b^	9.95 ± 0.11^b^	65.00 ± 6.01^a^	66.55 ± 2.29^b^	69.09 ± 2.57^ab^
YSG	7.08 ± 0.10^c^	9.58 ± 0.20^c^	9.02 ± 0.18^c^	49.09 ± 2.97^b^	65.45 ± 5.35^b^	68.18 ± 5.65^b^

Means within the same column followed by the same lowercase letters are not significantly different (*P* < 0.05) as compared by the LSD test.

**Table 6:** Central composite design of the three variables and their corresponding experimental data.

Run	*X*_1_: sucrose (g/L)	*X*_2_: yeast extract (g/L)	*X*_3_: mineral (g/L)	Mycelial inhibition (%)	
				Observed	Predicted
1	1	−1	−1	78.32	75.15
2	−1	−1	−1	76.38	77.30
3	1	1	−1	81.06	80.25
4	−1	1	−1	93.73	91.41
5	1	−1	1	81.67	82.03
6	−1	−1	1	82.86	81.71
7	1	1	1	89.46	86.58
8	−1	1	1	94.07	95.28
9	−1.68	0	0	76.46	79.38
10	1.68	0	0	88.63	88.49
11	0	−1.68	0	74.98	75.84
12	0	1.68	0	89.63	91.54
13	0	0	−1.68	78.86	81.12
14	0	0	1.68	89.64	90.16
15	0	0	0	92.48	96.06
16	0	0	0	97.67	96.06
17	0	0	0	97.86	96.06
18	0	0	0	96.16	96.06
19	0	0	0	94.03	96.06
20	0	0	0	98.65	96.06

**Table 7:** Analysis of variance (ANOVA) for the quadratic model.

Source	SS	DF	MS	*F*-value	*P* value
Model	1155.73	9	128.41	16.93	<0.0001^*∗*^
*X*_1_	100.23	1	100.23	13.21	0.0046^*∗*^
*X*_2_	297.38	1	297.38	39.20	<0.0001^*∗*^
*X*_3_	98.62	1	98.62	13.00	0.0048^*∗*^
*X*_1_ *X*_2_	40.64	1	40.64	5.36	0.0432^*∗*^
*X*_1_ *X*_3_	3.04	1	3.04	0.40	0.5410
*X*_*2*_ *X*_*3*_	0.15	1	0.15	0.020	0.8915
*X*_1_^2^	265.10	1	265.10	34.95	0.0001^*∗*^
*X*_2_^2^	275.69	1	275.69	36.34	0.0001^*∗*^
*X*_3_^2^	195.82	1	195.82	25.81	0.0005^*∗*^
Residual	75.86	10	7.59		
Lack of fit	46.41	5	9.28	1.58	0.3149
Pure error	29.45	5	5.89		
Total	1231.59	19			

*R* ^2^ = 0.9384; ^*∗*^significant at *P* value less than 0.05.

## Data Availability

The data sets analyzed during the current study are available from the corresponding author on reasonable request.
